# Forest Soil Phosphorus Resources and Fertilization Affect Ectomycorrhizal Community Composition, Beech P Uptake Efficiency, and Photosynthesis

**DOI:** 10.3389/fpls.2018.00463

**Published:** 2018-04-13

**Authors:** Aljosa Zavišić, Nan Yang, Sven Marhan, Ellen Kandeler, Andrea Polle

**Affiliations:** ^1^Forest Botany and Tree Physiology, University of Göttingen, Göttingen, Germany; ^2^Institute of Soil Science and Land Evaluation, Soil Biology, University of Hohenheim, Stuttgart, Germany; ^3^Laboratory for Radio-Isotopes, University of Göttingen, Göttingen, Germany

**Keywords:** fungal assemblage, nutrient stress, plant–microbe interaction, phosphorus nutrition, storage

## Abstract

Phosphorus (P) is an important nutrient, whose plant-available form phosphate is often low in natural forest ecosystems. Mycorrhizal fungi mine the soil for P and supply their host with this resource. It is unknown how ectomycorrhizal communities respond to changes in P availability. Here, we used young beech (*Fagus sylvatica* L.) trees in natural forest soil from a P-rich and P-poor site to investigate the impact of P amendment on soil microbes, mycorrhizas, beech P nutrition, and photosynthesis. We hypothesized that addition of P to forest soil increased P availability, thereby, leading to enhanced microbial biomass and mycorrhizal diversity in P-poor but not in P-rich soil. We expected that P amendment resulted in increased plant P uptake and enhanced photosynthesis in both soil types. Young beech trees with intact soil cores from a P-rich and a P-poor forest were kept in a common garden experiment and supplied once in fall with triple superphosphate. In the following summer, labile P in the organic layer, but not in the mineral top soil, was significantly increased in response to fertilizer treatment. P-rich soil contained higher microbial biomass than P-poor soil. P treatment had no effect on microbial biomass but influenced the mycorrhizal communities in P-poor soil and shifted their composition toward higher similarities to those in P-rich soil. Plant uptake efficiency was negatively correlated with the diversity of mycorrhizal communities and highest for trees in P-poor soil and lowest for fertilized trees. In both soil types, radioactive P tracing (H_3_^33^PO_4_) revealed preferential aboveground allocation of new P in fertilized trees, resulting in increased bound P in xylem tissue and enhanced soluble P in bark, indicating increased storage and transport. Fertilized beeches from P-poor soil showed a strong increase in leaf P concentrations from deficient to luxurious conditions along with increased photosynthesis. Based on the divergent behavior of beech in P-poor and P-rich forest soil, we conclude that acclimation of beech to low P stocks involves dedicated mycorrhizal community structures, low P reserves in storage tissues and photosynthetic inhibition, while storage and aboveground allocation of additional P occurs regardless of the P nutritional status.

## Introduction

In temperate forest ecosystems nutrient availability is one of the most important drivers for tree growth and ecosystem functions (Elser et al., 2007). Soils vary considerably in plant-available phosphorus (P) because rock-derived mineral P is depleted over geological time scales (Chadwick et al., 1999; Turner and Condron, 2013). These biogeochemical processes require shifts in plant nutritional strategies from mineral-based P supply to recycling of organic P (Foster and Bhatti, 2006; Lambers et al., 2008; Lang et al., 2016). Plants take up P in its inorganic form as phosphate (Becquer et al., 2014). Since inorganic P is usually depleted in soil solutions, P is often the first limiting macronutrient for plant growth under natural conditions (Vitousek et al., 2010). Plants can acclimate to low P availabilities by P remobilization and recycling (Maillard et al., 2015; Netzer et al., 2016; Zavišić and Polle, 2018) and by increasing P uptake efficiency, for instance, by increasing activities and affinities of P transporters (Kavka and Polle, 2016; Zhang et al., 2016). Thereby, a relatively higher fraction of P is acquired by P-deficient plants from the available pool of P in the soil than by P-sufficient plants (Koide, 1991; Sattelmacher et al., 1994; Schmidt et al., 2015).

Beech (*Fagus sylvatica* L.) forests are the most important forest type in Central European Lowland (Leuschner and Ellenberg, 2017). As the potentially natural vegetation they are of immense ecological significance (Leuschner and Ellenberg, 2017). Beech trees produce valuable timber and are therefore also of economic relevance (von Wühlisch and Muhs, 2011). Beech forests occur across a wide range of soil types differing in P supply (Peters, 1997; Leuschner et al., 2006). Studies of foliar P concentrations in different European countries did not indicate excess supply in any of the study locations and even revealed decreasing trends over longer temporal scales (Duquesnay et al., 2000; Ilg et al., 2009; Jonard et al., 2015; Talkner et al., 2015). To assess tree nutrition, foliar element concentrations are commonly used (Mellert and Göttlein, 2012). Since P concentrations in beech leaves vary considerably during the growth season, often resulting in similar levels in the foliage of trees stocking on P-poor and P-rich soil types (Haußmann and Lux, 1997; Netzer et al., 2016; Zavišić and Polle, 2018), the evaluation of the nutritional status is difficult. Altogether, these observations call for a better understanding of how beech trees acclimate to low soil P resources.

Mycorrhizal fungi are key to tree nutrition (Alvarez et al., 2009; Plassard and Dell, 2010; Cairney, 2011; Becquer et al., 2014; Johri et al., 2015). Therefore, an obvious idea is that mycorrhizal diversity might be affected by soil P stocks similar as known for nitrogen (Lilleskov et al., 2002; Cox et al., 2010). However, P fertilization studies in tree plantations did not show clear effects on the ectomycorrhizal communities (Treseder, 2004). Field studies along natural P gradients neither uncovered clear relationships between mycorrhizal diversity and P stocks (Twieg et al., 2009; Horton et al., 2013; Teste et al., 2016; Zavišić et al., 2016) although the fungal assemblages varied considerably among site conditions. Since divergent climatic and chemical soil factors also influence fungal communities, the elucidation of causal effects is difficult in ecological studies.

To overcome these shortcomings and to gain additional knowledge on the physiological differences between beech trees acclimated to low or high P supply, we conducted a common garden experiment, in which trees from a P-rich and a P-poor forest were fertilized with triple superphosphate (TP). The trees were excavated with intact soil cores in two beech forests differing in soil P stocks (Zavišić et al., 2016; Lang et al., 2017) and kept under ambient conditions from fall of the sampling year to the following summer in the presence or absence of fertilizer. By the comparison of fertilized trees and non-fertilized controls, we disentangled the effects of P on free living soil microbes, mycorrhizal communities colonizing the roots, plant P uptake efficiency, P contents, and photosynthesis. By applying ^33^P as a tracer, instantaneous plant uptake efficiency and P allocation were determined. Specifically, we addressed the following hypothesis: (i) P fertilization leads to increases in microbial biomass if microbes were P limited. We expected that an increase in microbial biomass might change belowground competition with roots for P. (ii) P fertilization causes shifts in the composition of mycorrhizal communities, regardless of soil types, if the communities were responsive to an increment in labile P (P_labile_). (iii) P fertilization affects P uptake efficiency more strongly in P-sufficient than in P-deficient plants because the latter have a higher P demand than well-supplied beech trees. (iv) P is preferentially allocated to leaves leading to enhanced photosynthesis. We expected no stimulation of photosynthesis in trees in P-rich soil, if their P supply was sufficient.

## Materials and Methods

### Plant Material and Triple Superphosphate Fertilizer Treatment

Beech seedlings (*Fagus sylvatica* L.) of ca. 0.4 m height were excavated in October 2014 in P-rich (Bad Brückenau, BBR) and in P-poor (Unterlüß, LUE) 120- and 137-year-old beech forests. The sites have been characterized in detail previously (Zavišić et al., 2016; Lang et al., 2017). Briefly, BBR is located in the natural biosphere reserve “Bayerische Rhön” (50°35′N, 9°92′E, 801–850 m above sea level. The climate is influenced by Atlantic air masses with an average long term sum of annual precipitation of 1000 mm, and a mean annual temperature of 5.8°C. The soil type is of volcanic origin (basalt), which is rich in minerals and nutrients. LUE is located in Lower Saxony (52°83′N, 10°36′E, 115 m above sea level) and has a mean annual temperature of 8.0°C, with an average annual precipitation of 730 mm. The soil type is sandy-loam with a low water capacity. A detailed description of the soils is given by Lang et al. (2017). Briefly, the soils of BBR/LUE contain 904/164 g P m^-2^, 1.3/0.7 kg nitrogen m^-2^, and 18/16 kg carbon m^-2^ to a depth of 1 m and have a pH value of 3.8/3.5 (Lang et al., 2017).

Young trees were collected inserting a polymer pipe (diameter 0.12 m, height: 0.2 m) into the soil around each individual and pulling it up with its intact soil core. The plants were exposed in a common garden experiment (Göttingen, 51°56′N, 9°96′E, 167 m above sea level) under ambient conditions under shading nets. The shading was necessary since all trees were collected in the understory. After 1 month acclimation (until November 20, 2014), half of the trees from each site were fertilized with TP (46% (w/w) P_2_O_5_, AGRAVIS Raiffeisen AG, Obernjesa, Germany) which is moderately soluble Ca(H_2_PO_4_)_2_ and typically used in agriculture for P-fertilization. TP was ground to a powder, sprinkled on the surface of the soil and subsequently dissolved by irrigation with tap water. Each tree obtained once a dose of 795 mg P. Control (CO) trees were irrigated with the same amount of tap water. These treatments resulted in the following experimental layout with 10 plants per treatment and site: CO beeches in P-poor soil, CO beeches in P-rich soil, TP beeches in P-poor soil, TP beeches in P-rich soil. The pipes, each containing plants of different treatments, were arranged in perforated crates and moved regularly to avoid positional effects. All trees were maintained under ambient conditions until harvest in the following summer in July and were watered regularly with tap water. For this experiment, a total of 40 plants were available (20 for radioactive treatments and 20 non-labeled controls).

### Determination of Photosynthesis

Gas exchange was measured on June 29, 2015 using seven plants from each site and treatment. Net photosynthetic rates, transpiration, stomatal conductance, and the sub-stomatal and ambient CO_2_ concentrations were determined using a portable photosynthesis system (LC Pro, ADC Bioscientific, Hoddesdon, United Kingdom). The light level was 350 μmol quanta m^-2^ s^-1^ of photosynthetically active radiation.

### Radioactive Labeling and Plant Harvests

Non-labeled TP and CO trees were harvested on 15^th^ and 16^th^ July 2015 (5 per treatment and site). The remaining trees (5 per treatment and site) were labeled with H_3_^33^PO_4_ (Hartmann Analytic GmbH, Braunschweig, Germany) to determine the P uptake rates. We used ^33^P because of its longer half-life time of 25.4 days, thus, resulting in higher signals than ^32^P (half-life time of 14.3 days) after 1 week of labeling. Further, the permitted limit of labeling outside of the control area (10^5^ kBq for the whole experiment) had to be considered. Here, we added 1.912 MBq in 40 ml of tap water to each plant, amounting to a total of 0.017 nmol P per plant. This corresponded to less than 10^-8^ of the P content in the soil and thus, had no effect on P availability. Each plant was subsequently watered with tap water to distribute the radioactive marker throughout the soil core. To avoid loss of label via through-flow, a plastic saucer was placed underneath each soil core. Flow-through after labeling was collected and re-applied to the soil core. All plants were irrigated with 40 ml tap water to avoid drought stress when required. During the labeling period mean air temperatures were 20.8 ± 0.5°C and the relative air humidity was 69.9 ± 1.4 %. The trees were harvested 7 days after ^33^P application. At harvest, the relative soil moisture across all treatments was 28 ± 6%.

### Harvesting Procedures and Microbial Biomass Determination

At the harvesting time, main stem heights and stem diameters at the base were measured. The basal stem of each tree was cut approx. 0.01 m above the soil. The soil was subsequently pushed out of the polymer smooth walled pipe, and the organic layer separated from the mineral topsoil. The masses of each soil layer and of all plant fractions (buds, leaves, stem, fine roots, and coarse roots) were recorded for each plant. Before separation of the root system, the roots were gently shaken and the soil adhering to the roots thereafter was considered as rhizosphere soil. The rhizosphere soil was collected from the roots by brushing them carefully with toothpicks, thereafter weighed, dried and used for chemical analyses. The roots were briefly washed and used immediately for mycorrhizal analyses. Aliquots of soil and plant tissues were dried at 40°C for 1 week to determine the dry mass. Further aliquots were kept frozen at -80°C.

To obtain wood and bark exudates, an about 2 cm long basal segment of the stem was cut and debarked. The bark and the peeled xylem sample were incubated for 5 h in a solution of 10 mM Na_2_EDTA (pH 7.0) and 15 mM chloramphenicol as described previously (Yang et al., 2016). The exudates were stored at -20°C.

Fresh soil samples were used for chloroform fumigation extraction and determination of microbial biomass (C_mic_ and N_mic_) as described previously (Spohn et al., 2018). Data were calculated with conversion factors of 0.45 for C_mic_ and 0.54 for N_mic_.

### Mycorrhizal Morphotyping and Species Identification

All fine root tips (<2 mm diameter) were inspected under a dissecting microscope (Leica M205 FA; Leica, Wetzlar, Germany) and classified according to different fungal morphotypes as described before (Zavišić et al., 2016; Spohn et al., 2018). All mycorrhizal root tips of a given plant were inspected. Aliquots of unknown morphotypes were used for DNA extraction and identification based on sequencing of the rRNA ITS-region (White et al., 1990) and alignment of the sequences with GenBank or UNITE. Species names were accepted when the sequence identity was >97% and the score >900 bits. Sequences were deposited in GenBank (MG820044-MG820051, KX168637, KX168639, KX168640, KX168642, KX168647, KX168650, KX168651, KX168655, KX168659, KX168660, KX168661, KX168663, KX168664, and KX168665).

### Determination of Labile and Total Phosphorus

For extraction of P_labile_ about 100 mg of dried, milled soil was used. The samples were suspended in 10 ml Bray-1 solution (1 M NH_4_F, 0.5 M HCl) and shaken for 5 min at 180 rpm. The samples were afterwards filtered using phosphate free filter paper (MN 280¼, Macherey-Nagel, Düren, Germany). The filtrate was analyzed colorimetrically at 645 nm wavelength (Specord 205, Analytik Jena, Germany) using malachite green oxalate (Sigma, St. Louis, MO, United States) as reagent according to the procedure described by Lanzetta et al. (1979).

Total P (P_tot_) was extracted from about 50 mg of dried, milled soil and plant material in 2 ml 65% HNO_3_ at 160°C for 12 h according to Heinrichs et al. (1986). Extracted samples were filtered using phosphate free filter paper (MN 280¼, Macherey-Nagel, Düren, Germany), and used for elemental analysis by inductively coupled plasma–optical emission spectroscopy (ICP–OES) (iCAP 6000 Series ICP–OES, Thermo Fisher Scientific, Dreieich, Germany). Bark and xylem exudates were directly used after filtration for P determinations.

### Determination of ^33^P in Soil and Plant Tissues

Extracts used for P_tot_ determination and bark and xylem exudates were used for measuring ^33^P. Three ml of extract from each plant tissue and soil layer were mixed with 10 ml of scintillation cocktail (Rotiszint eco plus, Roth, Karlsruhe, Germany) and for detection of radioactivity in a Perkin-Elmer scintillation counter (Tri-Carb TR/SL 3180, Waltham, MA, United States). The non-labeled samples were also measured as controls and showed no background signal. The ^33^P signal was corrected using QuantSmart (version 4.00, PerkinElmer) for its half-life of 25.34 days.

### Calculations and Statistical Analyses

We refer to P (mg g^-1^ dry mass) as P concentration and to the amount of P (mg) in a given soil compartment or tissue as P content.

Whole-plant P content was determined as

Whole-plant P content =                                                    Pbud (mg g-1) × biomass of buds (g) + Pleaf (mg g-1) ×          biomass of leaves (g) + Pstem (mg g-1) × biomass of stem (g)+ Pcoarse roots (mg g-1) × biomass of coarse roots (g) +            Pfine roots (mg g-1) × biomass of fine roots (g).                      

Whole-plant ^33^P (Bq) was determined correspondingly with the tissue dry masses and their ^33^P (Bq g^-1^ dry mass) concentrations.

P content of the soil per soil core was determined as

P content of the soil =                                                  Porganic layer (mg g-1) × dry mass of organic layer (g)         + Pmineral top soil (mg g-1) × dry mass of mineral top soil (g)+ P Rhizosphere (mg g-1) × dry mass of rhizosphere soil (g).

^33^P content of soil was determined correspondingly. The labile ^33^P content of the soil was calculated as:

Soil 33Plabile content (Bq) = Plabile content of soilPtot content of soil × 33P content of soil

Since P uptake rates of beech vary in different seasons (Zavišić and Polle, 2018), we distinguished between instantaneous uptake in summer determined 1 week after ^33^P application as whole-plant ^33^P content on the one hand and whole-plant P content as the result of life-time net P accumulation on the other hand. These measures were used to determine P uptake efficiencies according to Lang et al. (2017):

Instantaneous P uptake efficiency = whole-plant 33P contentsoil33Plabile content

P uptake efficiency = whole-plant P contentwhole soil Plabile content

The plant P uptake rate was calculated as:

P uptake rate (mg week-1) = whole-plant 33P content after

1 week/(specific activity) with the following estimate:

Specific activity (Bq mg-1 P) = Soil 33Plabile content (Bq)Plabile content of soil core

Data are shown as means (± SE). Data were analyzed by ANOVA followed by comparisons of means with Tukey HSD (package: “multcomp”) using R version 2.9.1 (R Core Development Team, 2012). Differences between means were considered to be significant when *p* ≤ 0.05. Testing for homogeneity of variances and normal distribution was done by analyzing residuals of the models and performing a Shapiro-Wilk test. When the data violated the assumption of normal distribution, data were log-transformed, before ANOVA was performed. Correlation coefficients were calculated using Pearson moment product.

Diversity indices (Shannon, Dominance, Pielou’s Evenness), ordination by non-metric multidimensional scaling (NMDS) and analysis of similarities (ANOSIM) were calculated for mycorrhizal assemblages using PAST software package 3.08^1^ (Hammer et al., 2001). Because all ectomycorrhizal root tips of a plant were counted the sample sizes varied. Therefore, the Raup-Crick method for presence–absence data was employed for calculation of dissimilarities since this method can handle variable sample sizes.

## Results

### Fertilization Increases the Labile Fraction of P in Soil but Has No Effect on Microbial Biomass

The P-poor soil contained the lowest concentrations of P_tot_ and P_labile_ in the organic layer as well as in the mineral top soil (**Table 1**). Fertilization resulted in significant increases in P_labile_ and P_tot_ in the organic layer in both the P-rich and the P-poor soil. In the mineral top soil, only the P-rich site exhibited higher P_labile_ concentrations after fertilization, whereas the increment in mineral soil from the P-poor sites was not significant (**Table 1**). P_tot_ concentrations in the mineral soil and in the rhizosphere were not affected by fertilization treatment (**Table 1**). Overall, fertilization resulted in about 3-times higher P_tot_ contents (per soil core) in P-poor soil than in non-fertilized control soil, whereas the P addition had no significant influence on P_tot_ content in the P-rich soil core (**Table 1**). However, in both soil types, fertilization increased the fraction of P_labile_ (**Table 1**).

**Table 1:** Phosphorus concentrations and microbial biomass in control and fertilized soil from a P-rich and a P-poor forest.

Parameter	Control		P-fertilized	
	P-rich	P-poor	P-rich	P-poor
P_labile_ in OL (mg g^-1^)	0.497 ± 0.118b	0.013 ± 0.003a	2.448 ± 0.122d	1.146 ± 0.054c
P_tot_ in OL (mg g^-1^)	1.673 ± 0.219b	0.211 ± 0.039a	2.096 ± 0.113d	1.149 ± 0.123c
P_labile_ in ML (mg g^-1^)	0.546 ± 0.101b	0.023 ± 0.001a	0.897 ± 0.088c	0.132 ± 0.028a
P_tot_ in ML (mg g^-1^)	1.432 ± 0.068b	0.069 ± 0.014a	1.515 ± 0.081b	0.157 ± 0.036a
P_tot_ in Rhizo (mg g^-1^)	1.385 ± 0.081b	0.151 ± 0.040a	1.885 ± 0.181b	0.614 ± 0.267a
P_tot_ content in soil core (mg)	1262 ± 186c	162 ± 27a	1482 ± 121c	510 ± 38b
P_labile_/P_tot_ in soil	0.35 ± 0.05b	0.21 ± 0.04a	0.74 ± 0.04c	0.97 ± 0.04c
C_mic_ in OL (μg g^-1^)	1685 ± 114b	948 ± 89a	1572 ± 211b	1069 ± 93a
N_mic_ in OL (μg g^-1^)	300 ± 35c	177 ± 14a	281 ± 36bc	203 ± 39ab
C_mic_ in ML (μg g^-1^)	628 ± 61b	196 ± 42a	607 ± 73b	84 ± 17a
N_mic_ in ML (μg g^-1^)	101 ± 12b	27 ± 4a	107 ± 9b	18 ± 2a

*Young beech trees in intact soil cores from the P-rich site (BBR, Bad Brückenau) and the P-poor site (LUE, Unterlüß) were fertilized once with 795 mg P in the form of TP or kept without fertilization (control) for 8 months in a common garden experiment. Soil P concentrations were measured at the final harvest [22^*nd*^ (P-rich) and 23^*rd*^ July 2015 (P-poor)] (n = 5, ± SE) and microbial biomass of carbon and nitrogen (C_*mic*_ and N_*mic*_) twice (15th/16th and 22^*nd*^/23^*rd*^ July 2015, n = 10 ± SE). Concentrations are expressed on the basis of soil dry mass. OL, organic layer, ML, mineral top soil layer, Rhizo, rhizosphere soil. Different letters indicate significant differences between means (Tukey’s HSD pairwise comparisons) at p < 0.05.*

The P-rich soil contained higher microbial biomass (C_mic_ and N_mic_) than the P-poor soil (**Table 1**). In both soil types, microbial biomass was higher in the organic layer than in the mineral top soil (*t*-test, *p* < 0.001). Fertilization had no significant effects on microbial biomass (**Table 1**).

### Mycorrhizal Community Structures of Beech in P-Poor Soil Are Influenced by P Fertilization

**FIGURE 1:**
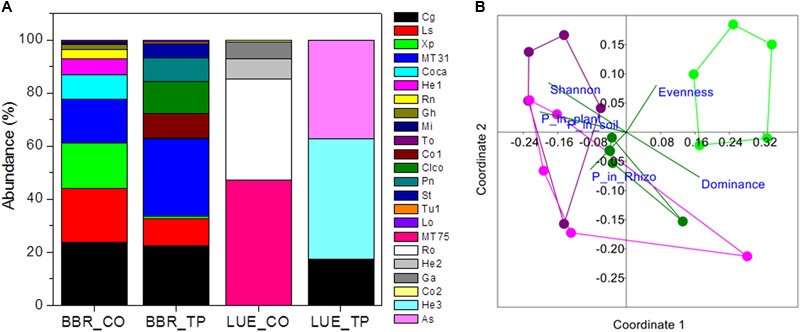
Relative abundance of ectomycorrhizal fungal taxa colonizing root tips of beech trees in P-rich (BBR) or P-poor soil (LUE) without [Control (CO)] or with application triple superphosphate (TP) fertilizer **(A)** and non-metric multidimensional scaling (NMDS) of the fungal assemblages **(B)**. Stress: 0.194. Vectors indicate explanatory variables with the diversity indices (Shannon H, Evenness, and Dominance) and P contents (whole-plant P content, P content of soil, and P content of rhizosphere). Symbols refer to ectomycorrhizal communities from BBR_CO: pink, BBR_FP: purple, LUE_CO: light green, LUE_FP: dark green. In **(A)** abbreviations refer to the following fungal taxa: Cg, *Cenococcum geophilum*; Ls, *Lactarius subdulcis*; X*p, Xerocomellus pruinatus*, MT31; Coca, *Cortinarius casimiri*; He1, *Helotiales* sp. 1; Rn, *Russula nigricans*; Gh, *Genea hispidula*; Mi, *Melanogaster intermedius*; To, *Tomentellopsis* sp.; Co1, *Cortinarius* sp_1.; Clco, *Clavulina coralloides*; Pn, *Pachyphlodes nemoralis*; St, *Solicoccozyma terricola*; Tu1, *Tuber* sp.; LO, *Leptodontidium orchidicola*, MT57; Ro, *Russula ochroleuca*; He2, *Helotiales* sp. 2; Ga, *Genea* cf. *anthracina*; Co2, *Cortinarius* sp. 2; He3, *Heliotales* sp. 3; As, Ascomycota; MT, morphotypes, for which sequencing failed.

In total 16 fungal morphotypes were detected, of which 14 were identified by ITS sequencing (**Figure 1A**). The number of taxa differed among the sites and was higher on trees in P-rich than in P-poor soil (**Table 2**). *Cenococcum geophilum* was absent on roots of trees in P-poor soil but was an abundant species in P-rich soil (**Figure 1A**). *C. geophilum* also appeared after P fertilization on roots of beech trees in P-poor soil (**Figure 1A**). Further abundant taxa that appeared after fertilization of P-poor trees were an unknown ascomycete and *Cortinarius casimiri* (**Figure 1A**). In P-rich soil, fertilization also resulted in changes; among the abundant fungi *Lactarius subdulcis* and *Xerocomellus pruinatus* decreased and *Clavulina coralloides, Leptodontidium orchidicola* and an unspecified *Cortinarius* sp. 1 increased (**Figure 1A**). Shannon indices were higher and Dominance indices of the mycorrhizal communities lower in P-rich than in P-poor soil (**Table 2**).

**Table 2:** Diversity indices for mycorrhizal assemblages colonizing the roots beech trees from a P-rich (BBR) and a P-poor (LUE) site.

	Control		P-fertilized		p_(Treatment)_	p_(Treatment)_	p_(Sites)_	p_(Sites)_	
	P-rich	P-poor	P-rich	P-poor	P-poor	P-rich	TP	CO	
Taxa	11	5	12	3	0.012	0.001	0.001	0.001	
Shannon H	1.933	1.133	1.915	1.030	0.531	0.006	0.001	0.001	
Evenness	0.628	0.621	0.565	0.933	0.001	0.001	0.001	0.807	
Dominance	0.168	0.377	0.179	0.375	0.029	0.801	0.001	0.001	

*Half of the trees were fertilized with TP; the other half served as the controls (CO). P-values obtained by permutation tests are indicated for the following pairwise comparisons: fertilized compared with controls at BBR: p_*(Treatment)*_ BBR, fertilized compared with controls at LUE: p_*(Treatment)*_ LUE, fertilized soil from BBR with fertilized soil from LUE: p_*(Sites)*_ TP, control soil from BBR with control soil from LUE: p_*(Sites)*_ CO*.

Non-metric multidimensional scaling ordination showed strong dissimilarity of the ectomycorrhizal communities from P-poor controls compared with those from the P-rich site (ANOSIM, controls: *p* < 0.05, fertilized: *p* < 0.01) as well as those from those in P-poor soil after fertilization (ANOSIM, *p* < 0.01, **Figure 1B**). Fertilization of the P-rich soil did not change the ectomycorrhizal communities’ structures (**Figure 1B**, ANOSIM, *p* = 0.385). The differences among the mycorrhizal communities in P-rich and P-poor soil were explained by higher Shannon diversity and higher P contents in soil, rhizosphere and plants for the ectomycorrhizal assemblages under P-rich conditions and by higher Evenness and dominance for the ectomycorrhizal assemblages in P-poor soil (**Figure 1B**).

### P Nutrition and Uptake Efficiency in Relation to Mycorrhizal Diversity

Application of P fertilizer resulted in higher plant P contents in both, beech trees in P-poor and P-rich soil (**Table 3**). P fertilization increased P tissue concentrations of plants in P-poor soil but had no significant effects on those in P-rich soil (**Table 3**). The P uptake efficiency was highest for P-poor control beech trees and declined upon fertilization to levels similar to those of P-rich plants (**Table 3**).

**Table 3:** Phosphorus contents, concentrations and uptake in beech trees grown control and fertilized soil from a P-rich and a P-poor forest.

	Control		P-fertilized	
	P-rich	P-poor	P-rich	P-poor
**P content**				
Whole-plant P_tot_ content (mg plant^-1^)	6.7 ± 1.2b	1.3 ± 0.2a	10.9 ± 1.6c	7.3 ± 0.5b
**P concentrations**				
Mean whole-plant P_tot_ (mg g^-1^)	1.32 ± 0.17b	0.47 ± 0.05a	1.60 ± 0.21b	0.78 ± 0.07a
P_tot_ in fine roots (mg g^-1^)^∗^	1.03 ± 0.06b	0.55 ± 0.03a	1.46 ± 0.28b	1.97 ± 0.09b
P_tot_ in coarse roots (mg g^-1^)^∗^	0.88 ± 0.14b	0.26 ± 0.02a	1.46 ± 0.16c	1.82 ± 0.33c
P_tot_ in stem (mg g^-1^)	0.84 ± 0.08b	0.26 ± 0.03a	1.12 ± 0.10b	0.98 ± 0.16b
P_tot_ in leaves (mg g^-1^)	1.15 ± 0.04ab	0.68 ± 0.06a	1.38 ± 0.11b	2.82 ± 0.33c
P_tot_ in buds (mg g^-1^)	1.02 ± 0.26a	1.09 ± 0.09a	1.43 ± 0.08a	2.54 ± 0.20b
P_tot_ in bark (mg g^-1^)	0.64 ± 0.02a	0.38 ± 0.02a	1.37 ± 0.22b	1.51 ± 0.30b
P in bark exudate (mg g^-1^)	0.15 ± 0.03a	0.09 ± 0.02a	1.04 ± 0.34b	1.38 ± 0.25b
P_tot_ in xylem (mg g^-1^)	0.84 ± 0.13b	0.16 ± 0.02a	1.36 ± 0.14c	1.11 ± 0.17bc
P_tot_ in xylem exudate (mg g^-1^)	0.040 ± 0.006b	0.011 ± 0.002a	0.067 ± 0.005c	0.075 ± 0.012c
**P uptake**				
P uptake efficiency (plant^-1^)^∗^	0.018 ± 0.005a	0.047 ± 0.009b	0.010 ± 0.001a	0.015 ± 0.001a
Instant. P uptake efficiency (plant^-1^)^∗^	0.016 ± 0.003b	0.069 ± 0.023c	0.006 ± 0.001a	0.005 ± 0.001a
Whole-plant P uptake rate (mg week^-1^)^∗^	7.86 ± 2.78b	2.08 ± 0.77a	6.27 ± 1.32b	2.42 ± 0.28ab
P uptake rate (mg g^-1^ week^-1^)	1.52 ± 0.49b	0.79 ± 0.32ab	0.97 ± 0.25b	0.25 ± 0.03a

*Young beech trees in intact soil cores from the P-rich site (BBR, Bad Brückenau) and the P-poor site (LUE, Unterlüß) were fertilized with TP or kept without fertilization for 8 months in a common garden experiment. (n = *5, ± SE) Different letters in rows indicate significant differences between means (Tukey’s HSD pairwise comparisons) for p < 0.05. For the definitions and calculations of P uptake efficiency, instantaneous (inst.) P uptake efficiency and P uptake rates refer to section “Materials and Methods*”. ^∗^Data were log-transformed for statistical analysis.*

Since the uptake efficiency for P_tot_ integrates over the whole life history of the plants, we were also interested in testing whether the results would hold for current P uptake. To determine instantaneous P uptake efficiency, ^33^P was added as a tracer to the soil. Similar to the P uptake efficiency, the instantaneous P uptake efficiency was highest in control plants in the P-poor soil and lower in plants in P-rich soil and after fertilization (**Table 3**). Moreover, a strong decline in the instantaneous P uptake efficiency became apparent in fertilized plants resulting in about 10-fold lower values than those found for the P-poor conditions (**Table 3**). Despite lower uptake efficiencies, the estimated P uptake rates were generally higher for plants in P-rich than in P-poor soil and not significantly influenced by fertilization (**Table 3**).

**FIGURE 2:**
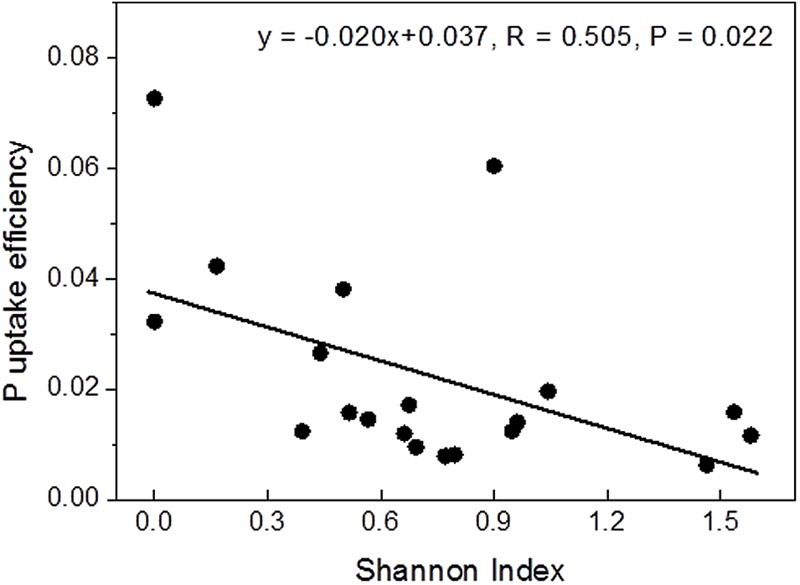
Relationship between Shannon diversity of the mycorrhizal root tips and whole-plant P uptake efficiency.

Correlation analyses were conducted to test whether P contents or uptake were related to mycorrhizal diversity. Among the tested variables, Shannon diversity showed a strong linear negative correlation with P uptake efficiency (**Figure 2**), while the relationship with instantaneous P uptake efficiency was less strong (*R* = -0.455, *P* = 0.044) and those with whole plant P content (*R* = 0.433, *P* = 0.056) and P uptake rate (*R* = 0.393, *P* = 0.086) were not significant (Supplementary Figure S1).

### P Fertilization Rescues Photosynthesis of P-Limited Beech Through Preferential Above-Ground Allocation

**FIGURE 3:**
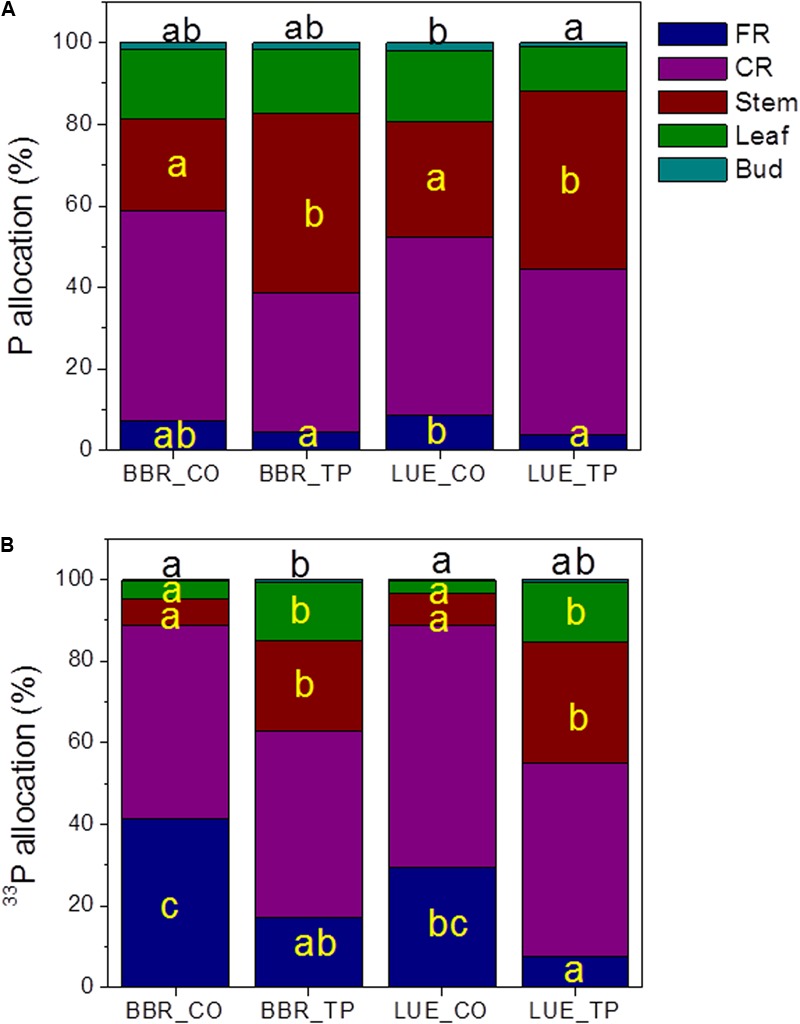
Whole-plant P_tot_ **(A)** and ^33^P **(B)** allocation in European beech trees (*Fagus sylvatica* L.) in P-rich (BBR) or P-poor soil (LUE) without (CO) or with application of TP fertilizer. The contents of P (mg) and of ^33^P (Bq) were determined for each tissue 1 week after application of the radioactive label. For each plant, the sum of the tissue P_tot_ contents, respective the sum of the ^33^P contents was set as 100%. Data indicate means (*n* = 5 per soil type and P treatment). Significant differences among the tissues at *p* < 0.05 are indicated by different letters. Black letters on top of the stacked bars refer to buds. Tissues without letters did not show any significant difference.

To investigate how beech plants handle the distribution of limiting or abundant P across different tissues, we determined the allocation of whole-plant P_tot_ and newly taken up P (^33^P). The majority of whole-plant P_tot_ was present in coarse roots and stems (**Figure 3A**), while a large fraction of new P was also found in fine roots (**Figure 3B**). Fertilization shifted P_tot_ and ^33^P allocation to stem tissue (**Figure 3A**).

Since the stem is both, transport and storage tissue, we wondered whether the additional P was mainly used for improved P supply to green tissues or indicated stronger accumulation of P reserves. Therefore, P concentrations in xylem and bark and in their respective exudates were determined. Both xylem and xylem exudates showed increased P concentrations after fertilization indicating higher transport (**Table 3**), but the relative P fraction in the xylem exudates was unaffected by fertilization amounting 6 ± 1% of the P concentration in the xylem. This finding indicates increased P storage. The bark concentrations of P also increased in response to fertilization (**Table 3**) but in this case, the fraction of P in the exudates also strongly increased from about 25% in controls to about 70% (P-rich) and 90% (P-poor) in fertilized plants (*p* < 0.01) suggesting enhanced P circulation in the fertilized compared with the control plants.

The P_tot_ concentrations in leaves and buds of the plants in P-poor soil increased drastically in response to fertilization, whereas no significant changes in P_tot_ were found for the fertilized plants in P-rich soil (**Table 3**). However, regardless the soil type, the fraction of new ^33^P was enhanced in leaves of fertilized compared with those of control plants (**Figure 3B**).

**FIGURE 4:**
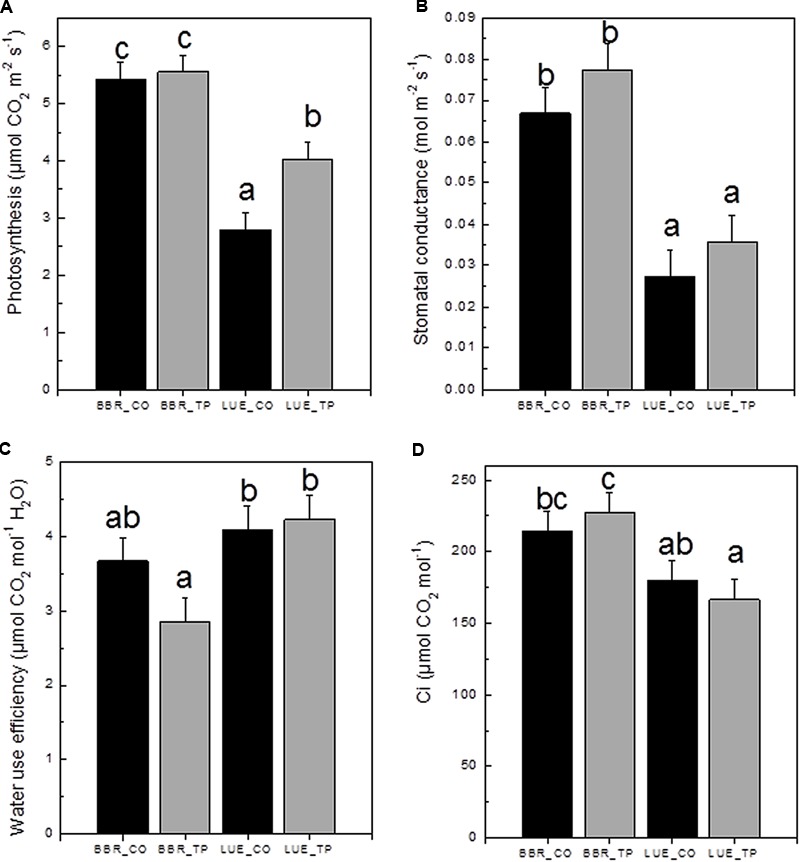
Net photosynthetic rates **(A)**, stomatal conductance **(B)**, water use efficiency **(C)**, and substomatal CO_2_ concentration c_i_ **(D)** of European beech trees (*Fagus sylvatica* L.) in P-rich (BBR) or P-poor soil (LUE) without (CO, black bars) or with application of triple superphosphate fertilizer (TP, gray bars). The trees were exposed in a common environment outdoors in October 2014 and fertilized in November 2014. The measurements were performed on June 29, 2015. Values for air temperature, relative air humidity, and ambient CO_2_ concentrations during the times of the measurement were as follows: LUE_TP: 26.3°C, 69.3%, 379 ppm; LUE_CO: 27.1°C, 71.5%, 378 ppm; BBR_TP: 30.3°C, 64.7%, 379 ppm; BBR_CO: 30.4°C, 75.3%, 380 ppm. Bars represent means ± SE (*n* = 7). Different letters indicate significant differences at *p* ≤ 0.05.

To investigate the physiological consequences of P fertilization, photosynthetic gas exchange was measured. CO_2_ assimilation was higher in plants from the P-rich than in plants from the P-poor site (**Figure 4A**). Fertilization resulted in 60% increased photosynthesis rates in plants from the P-poor site but had no effect on plants from the P-rich site (**Figure 4A**). One reason for lower photosynthesis of P-poor plants was their lower stomatal conductance (**Figure 4B**). This reduction was not caused by water limitations because the overall soil moisture (28 ± 6%) in the soil cores did not differ among the treatments and soil types (*p* > 0.05). Limitations in CO_2_ consumption can also lead to decreases in stomatal conductance as the result of increased substomatal CO_2_ concentrations (Damour et al., 2010). However, this was not the case here (**Figure 4D**). Beech trees from the P-poor site exhibited higher water use efficiencies than fertilized trees from the P-rich site (**Figure 4C**).

## Discussion

### Impact of P Fertilization on Soil Microbes and Mycorrhizal Communities

About 8 months after application of P fertilizer and regular irrigation of the plants with tap water, 77% (P-rich) and 64% (P-poor) of the added P had disappeared. Since we determined the P contents for all compartments in our experimental system and other leaks are unknown, the losses were most likely caused by leaching. Still, the concentrations of P_labile_ in the organic layer of P-fertilized soil were higher than those in non-treated soil, indicating persistent effects (**Table 1**). Since P is exchanged between microbial biomass and soil solution (Achat et al., 2010, 2012) microbial activities may influence tree nutrition. Seasonal experiments with soil cores in a similar set-up as in the present study showed that a large fraction of P was bound in microbial biomass and, thus, not available for plant nutrition (Spohn et al., 2018). Therefore, a critical point in the current study was to clarify whether P concentrations resulted in enhanced microbial biomass. Here, no significant changes in microbial biomass were observed in response to P fertilization but microbial biomass was generally lower in P-poor than in P-rich soil (**Table 1**). A reduction of microbial biomass in P-poor compared to P-rich soil has also been reported in previous studies conducted in the forests at BBR and LUE (Zavišić et al., 2016; Zederer et al., 2017). Since we did not find an amelioration of microbial biomass in response to P fertilization, other nutrients such as carbon might have limited microbial biomass (Achat et al., 2012; Heuck et al., 2015). Lower carbon assimilation of plants in P-poor soil, as observed here (**Figure 4A**), would be expected to diminish belowground carbon flux and thereby, might limit microbial biomass carbon. Consequently, higher plant assimilation and biomass production in response to P additions could have positive feed-back effects on microbial biomass in the long run. Alternatively, microbial growth might also be limited by nitrogen in our sites. Using respiration measurements under different carbon, nitrogen and P amendments, Preusser et al. (2017) revealed that beech forest soils site were initially carbon limited, followed by subsequent nitrogen limitation. Only when nitrogen limitations were overcome by N amendments, addition of P enabled a surplus of microbial growth response (Preusser et al., 2017).

Previous studies on beech roots from the old trees in the P-rich and P-poor forests revealed lower mycorrhizal species richness and compositional differentiation of the assemblages under low P availability (Zavišić et al., 2016). Here, we confirmed this observation for young beech trees from those sites (**Figure 1** and **Table 2**). Moreover, we clearly show that both diversity and composition were influenced by fertilization of P-poor beech trees (**Figure 1** and **Table 2**). This finding indicates that P availability is a driver for mycorrhizal fungal species composition in forest soil. The effect of fertilization on the mycorrhizal community was stronger in P-poor than in P-rich soil (**Figure 1B**). In P-rich soil, the fungal communities consisted of taxa typically found in beech forests such as *C. geophilum, L. subdulcis, X. pruinatus, Clavulina* sp., and *Cortinarius* sp. (Courty et al., 2010; Lang et al., 2011; Pena et al., 2017). In contrast to P-poor soil, P-fertilization did not result in significant shifts of the community structure in P-rich soil, despite some changes in abundances of fungal taxa (**Figure 1A**). To date, the few studies available on the influence of P fertilization on ectomycorrhizal communities have been conducted in plantations, also demonstrating P-induced changes in the communities (*Populus*: Baum and Makeschin, 2000; dipterocarps: Pampolina et al., 2002; *Eucalyptus*: Brearley et al., 2007). In our study, it was notable that only ascomycota were detected on the roots of fertilized trees in P-poor soil and that the abundance of this phylum also increased on roots of fertilized trees in P-rich soil (**Figure 1A**). These shifts might indicate the development of mycorrhizal communities, which are more typical for disturbed conditions (Smith and Bonito, 2012). Furthermore, mycorrhizas formed by Ascomycota do not form widely extending extramatrical hyphae (Smith and Bonito, 2012), thus, relying on nutrients in their immediate vicinity. Our results suggest that larger P availability may favor such shifts because the similarity of mycorrhizal community structures of fertilized beeches in P-poor soil was closer to those of beeches in P-rich than to non-fertilized beeches in P-poor soil (**Figure 1B**).

Ectomycorrhizal communities are composed of functionally different taxa (Courty et al., 2010; Pritsch and Garbaye, 2011; Rineau and Courty, 2011; Pena et al., 2013; Pena and Polle, 2014). Here, we found that plant P uptake efficiency was strongly negatively correlated with the diversity of the fungal assemblage (**Figure 2**). A reason for this unexpected result could be that the fungal taxa associated with P-poor plants were more specialized for P acquisition and uptake than the communities of diverse taxa that colonized the well-supplied plants. For example, the fungal assemblages after fertilization as well as those in P-rich soil contained a significant fraction of *C. geophilum*. This fungus has lower phosphatase activities than many other mycorrhizal fungal taxa (Baxter and Dighton, 2005). This fungal trait might be less important under conditions of a high availability of P_labile_. However, our suggestion that P limitation leads to functionally adapted, more specialized assemblages is speculative and should be tested in further P fertilization experiments combined with P uptake studies of distinct fungal taxa.

### Beech Responses to P Fertilization

In the presence of high P availability, plants down-regulate their P uptake systems decreasing their uptake efficiency (Nussaume et al., 2011; Baker et al., 2015; Kavka and Polle, 2016; Kavka and Polle, 2017). It is, therefore, conceivable that regulation of P transport contributed to lower P uptake efficiencies observed here for trees in P-rich compared with those in P-poor soil. As expected, fertilization resulted in further decreases but in contrast to our working hypothesis that fertilized trees in P-poor soil would respond with less reduction in uptake efficiency, they showed a 10-fold decrease while that of P fertilized trees in P-rich soil was only threefold reduced (**Table 3**). The estimated P uptake rate (per unit of biomass) also dropped to its lowest level in fertilized beech trees in P-poor soil. This observation is surprising at the first glance. However, P uptake is regulated by long-distance signaling in response to transcription factors that survey the cellular P status (Chiou and Lin, 2011; Scheible and Rojas-Triana, 2015). In our study, P concentrations in leaves of fertilized plants in P-poor soil were strongly increased, from deficient (0.7 mg P g^-1^ dry mass) to excessive levels (2.8 mg P g^-1^) according to the common classification scheme (P [mg g^-1^ dry mass]: deficient <1.1, normal 1.1–1.9, extreme >2.0, Mellert and Göttlein, 2012]. The fertilized trees on P-poor soil reached the highest foliar P concentrations among all conditions tested (**Table 3**). It is likely that this enrichment set off a regulatory cascade that shut down P uptake at the level of roots. The profound increase of P in bark exudate, which is a proxy for P transport (King and Zeevaart, 1974; Dambrine et al., 1995; Gessler et al., 1998; Yang et al., 2016), further supports this idea.

A word of caution is warranted regarding the estimated P uptake because the values are fairly high. In agreement with other studies (Morel and Fardeau, 1989; Bünemann et al., 2007; Oberson et al., 2010), we used the fraction of P_labile_ to determine the specific ^33^P activity, required to accommodate for differences in tracer dilution in soil. P_labile_ indicates the maximum P fraction available, whereas the actual P concentrations in soil solutions are lower (e.g., Zavišić et al., 2016) and, thus, must result in lower *de facto* fluxes than those calculated here. Since the “true” P concentrations in the soil volume of the root tips are unknown and may vary spatially depending on P uptake by different mycorrhizal taxa, refined analyses of uptake rates are currently not possible. It is obvious that more information on the activities of distinct mycorrhizal taxa in their natural assemblages for P uptake and the molecular biology of these processes is urgently needed to better understand P fluxes.

Our P fertilization experiment was instrumental to distinguish between deficient and sufficient P supply of the young field-grown beech trees because the plants in P-rich soil did not respond with increases in photosynthesis although they exhibited increased P contents, whereas the plants in P-poor soil showed a strong stimulation of photosynthetic CO_2_ assimilation (**Figure 4**). The positive response to P amendment is in agreement with many other studies showing that fertilization can increase photosynthetic rates in trees (Kirschbaum and Tompkins, 1990; Gough et al., 2004; Turnbull et al., 2007; King et al., 2008; Warren, 2011). Our results show that additional P taken up by fertilized trees in both soil types was preferentially allocated aboveground resulting in increased P accumulation in storage tissues (**Figure 3**). This is an important finding since P growth demand and P uptake are temporally decoupled in beech (Zavišić and Polle, 2018). P-deficient beech trees, as those studied her, develop a massive temporal P deficit during early growth, which is replenished in late summer and fall (Zavišić and Polle, 2018). In winter, almost no differences exist between the P nutritional status of P-deficient and P-sufficient trees (Zavišić and Polle, 2018). This balance is achieved by lower growth rates (Zavišić and Polle, 2018). In combination, those results and the current study highlight the importance of P storage pools and transport tissues for acclimation of beech to low P availabilities.

## Conclusion

P fertilization shaped mycorrhizal fungal assemblages, shifting the community composition on roots in P-poor soil to a higher similarity to those on roots in P-rich soil but did not affect microbial biomass. Higher fungal taxa diversity in P-rich soil correlated with lower plant P uptake efficiency, which might indicate a higher specialization of fungal taxa colonizing roots in a stressful than in a less stressful environment.

In nature, beech can form closed stands even on the poorest geological substrates, thus, demonstrating large ecological amplitude for nutritional conditions (Leuschner and Ellenberg, 2017). Therefore, the diagnosis of nutrient deficiencies is difficult. Here, we found that P amendment increased the P contents of both, trees in P-poor and P-rich soil but enhanced photosynthesis only in trees in P-poor soil, thus, pin-pointing that young trees in P-rich soil were sufficiently P supplied, whereas those in P-poor soil suffered from P deficiency. Altogether the current studies (Netzer et al., 2016; Yang et al., 2016; Spohn et al., 2018; Zavišić and Polle, 2018) show that beech has a high metabolic flexibility to cope with low soil P stocks by growth adjustment. Since P deficiency results in drought stress-like photosynthetic responses such as decreased stomatal conductance, decreased substomatal CO_2_ concentrations and a trend toward enhanced water use efficiency (Yang et al., 2016, this study), we suspect that P-deficient trees will be more vulnerable than P-sufficient trees when they encounter additional stresses in their long life span. Thus, climate change with longer periods of drought (IPCC, 2014) may endanger beech forests on P-limited soils. In future, it will be important to investigate the potential of nutrient-stressed beech trees to endure additional environmental constraints.

## Data Availability Statement

The raw data supporting the conclusions of this manuscript have been deposited in the central repositorium of Priority Program 1685 and will be made available by the authors, without undue reservation, to any qualified researcher.

## Author Contributions

AZ and AP contributed to the conception and design of the study. AZ, NY, and SM conducted the measurements and analyzed the data. AP and EK analyzed the data. AZ wrote the first draft of the manuscript. AP revised the draft. All authors contributed to manuscript revision and read and approved the submitted version.

## Conflict of Interest Statement

The authors declare that the research was conducted in the absence of any commercial or financial relationships that could be construed as a potential conflict of interest.

## Footnotes

1 https://folk.uio.no/ohammer/past/
