# Supplementary material for: Forest Soil Phosphorus Resources and Fertilization Affect Ectomycorrhizal Community Composition, Beech P Uptake Efficiency, and Photosynthesis
Source: Front Plant Sci. 2018 Apr 13;9:463. doi: 10.3389/fpls.2018.00463 (PMC5908982; doi:10.3389/fpls.2018.00463)
Supplement: Supplementary file 1 [file Presentation_1.PDF]

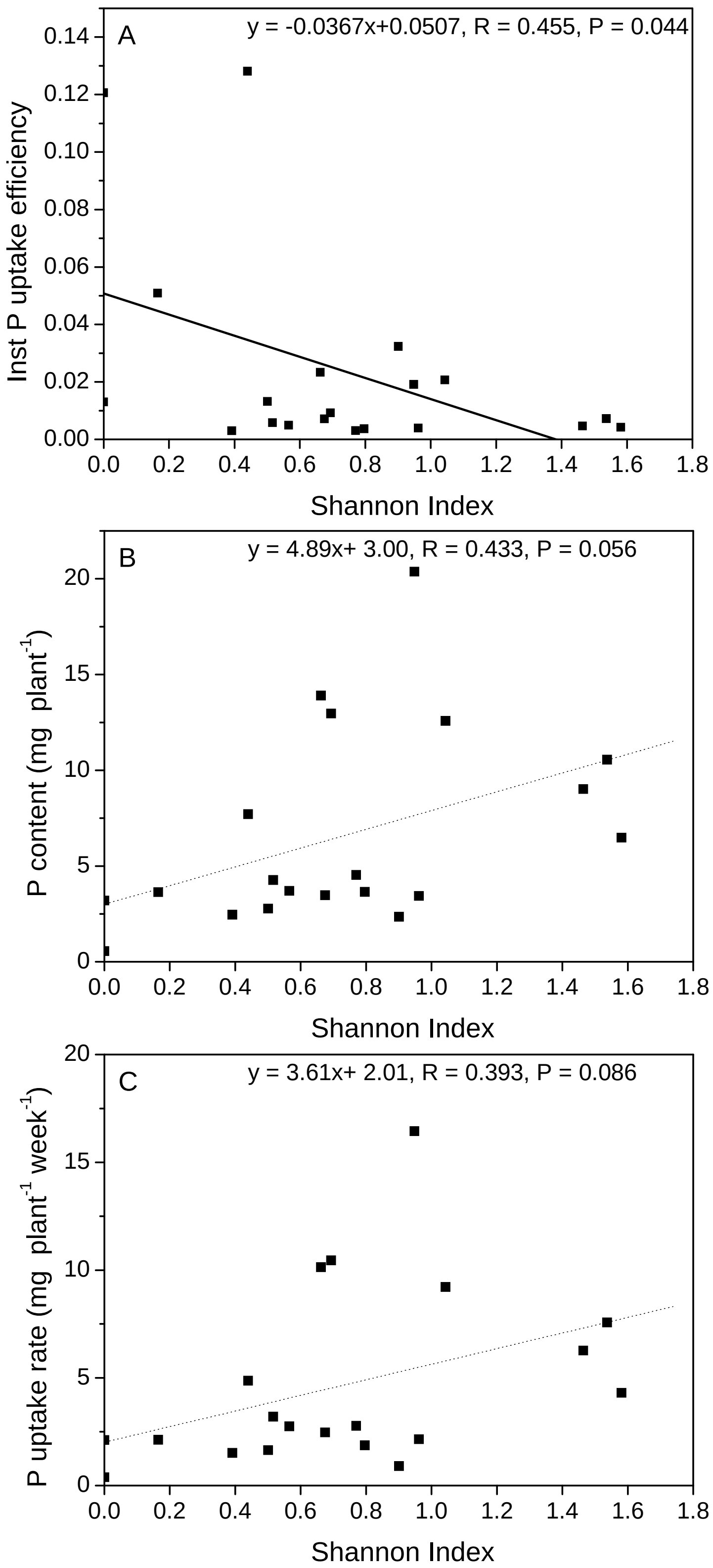

Figure S1. Relationships between Shannon diversity of the mycorrhizal root tips and instantaneous P uptake efficiency (A), whole-plant P content (B), and P uptake rate (C). When the correlations were not significant, broken lines are shown.
